# Predictive importance of cartilage acetabular index for acetabular dysplasia in orthopedic surgery

**DOI:** 10.1590/acb400625

**Published:** 2025-01-13

**Authors:** Mehmet Onur Ziyadanoğulları, Hüseyin Arslan

**Affiliations:** 1 Gazi Yasargil Research and Training Hospital – Department of Orthopedics and Traumatology – Diyarbakir – Turkey.; 2 Memorial Hospital – Department of Orthopedics and Traumatology – Diyarbakir – Turkey.

**Keywords:** Developmental Dysplasia of the Hip, Osteoarthritis, Arthrography, General Surgery

## Abstract

**Purpose:**

To investigate the relationship between the cartilage acetabular index and acetabular development and secondary dysplasia.

**Methods:**

A total of 58 hips underwent intraoperative arthrography-guided open reduction or limited open reduction due to developmental hip dysplasia between 2011 and 2015 was included in the study. We evaluated patients with acetabular angle 8º as group 2. Intraoperative acetabular cartilage index measurements were performed, and patients with low and high cartilage acetabular index were divided into two groups.

**Results:**

There was a correlation between the cartilage acetabular index value, which indicates preoperative cartilage acetabular coverage, and acetabular development and secondary acetabular development.

**Conclusion:**

The cartilage acetabular index is a technically easy and uncomplicated evaluation method that can be used to estimate acetabular development and should be used routinely together with the bone acetabular index.

## Introduction

Acetabular dysplasia (AD) is a common developmental disorder of the hip, characterized by an underdeveloped acetabulum that inadequately covers the femoral head, leading to joint instability and a predisposition to early-onset osteoarthritis. Early diagnosis and appropriate intervention are crucial to preventing long-term joint damage and improving functional outcomes in affected individuals. Traditional diagnostic tools, such as the acetabular index (AI), have been widely used to assess the severity of AD. However, emerging evidence suggests that the cartilage acetabular index (CAI) may offer a more sensitive and accurate measure, particularly in younger populations, in which the cartilaginous structures are predominant.

AD is a significant risk factor for hip dislocation and osteoarthritis, especially in young adults. The condition often remains asymptomatic until joint degeneration has progressed, making early diagnosis challenging, but essential for effective management^1^^,^ 2. The traditional AI, which measures the angle between a horizontal line through the triradiate cartilage and the acetabular roof, has been a cornerstone in diagnosing AD. However, this method primarily reflects the bony architecture of the hip, potentially overlooking the cartilaginous contribution to acetabular morphology, especially in pediatric patients^3^.

The CAI has emerged as a novel parameter that includes the cartilaginous acetabular rim in its assessment, providing a more comprehensive view of acetabular morphology. Unlike the AI, which is static and bony, the CAI dynamically reflects both bony and cartilaginous structures, making it particularly valuable in assessing younger patients^3^. The inclusion of the cartilaginous rim is crucial in early detection, as the cartilaginous components significantly contribute to hip stability during growth^4^.

Recent studies have highlighted the predictive value of the CAI in identifying patients at risk for developing AD and subsequent hip-related complications. For example, a longitudinal study by Sherman et al.^5^ found that an elevated CAI in infants was strongly associated with the development of AD and the need for surgical intervention later in life^5^. Additionally, Willemsen et al.^6^ demonstrated that the CAI could predict the progression of AD and the likelihood of osteoarthritis in adult patients, suggesting its potential as both a diagnostic and prognostic tool.

While the AI has been widely used for decades, its limitations, particularly in pediatric populations, have become increasingly apparent. The CAI, by incorporating the cartilaginous structures, may offer a more accurate assessment of acetabular development and dysplasia severity^7^. A comparative study by Schmaranzer et al.^8^ found that the CAI had superior sensitivity and specificity in detecting early AD compared to the traditional AI.

The clinical application of the CAI is still in its early stages, but its potential benefits are significant. Incorporating the CAI into routine clinical practice could enhance early detection of AD, allowing for earlier intervention and potentially better outcomes^9^. However, standardization of measurement techniques and the establishment of normative values across different age groups are necessary before widespread adoption can occur^10^.

Despite its promise, the CAI’s predictive accuracy and clinical utility remain subjects of ongoing research. A study by Wang et al.^11^ underscored the need for further longitudinal research to validate the CAI’s effectiveness in predicting long-term outcomes, such as the development of osteoarthritis or the need for hip replacement surgery. Moreover, there is a need to refine the CAI measurement techniques to reduce inter-observer variability and improve its reliability in different clinical settings^12^.

The CAI has gained significant attention as an important diagnostic tool for assessing AD, particularly when evaluated using anteroposterior radiography. The CAI measures the angle formed by the cartilaginous acetabular rim relative to the horizontal plane, providing critical insight into the development and alignment of the acetabulum. The anteroposterior (AP) radiograph is particularly valuable, because it offers a comprehensive view of the entire acetabulum and its relationship with the femoral head, enabling the detection of subtle dysplastic changes that might not be evident when using traditional osseous indices such as the AI or lateral center-edge angle (LCEA)^13^. This radiographic technique is often the first line of imaging in assessing dysplasia of the hip, offering a balance between accessibility, cost-effectiveness, and the ability to visualize both bone and cartilage structures. While conventional AI measurements primarily focus on bony landmarks, the CAI extends this analysis by incorporating the cartilaginous structure, making it particularly useful in younger patients whose bony development is not yet complete^14^.

Magnetic resonance imaging (MRI) is frequently employed alongside AP radiography to enhance the accuracy of CAI measurement, as it provides high-resolution images of both bone and cartilage, allowing for a more detailed assessment of the acetabular morphology^15^. This combined approach is critical, especially in cases in which early dysplastic changes are suspected, but not clearly visible on plain radiographs. Furthermore, the CAI measured in the AP view has been demonstrated to have strong predictive value for long-term outcomes in patients with AD. Studies have shown that an abnormal CAI in the AP view is strongly associated with the progression of dysplasia, leading to an increased risk of osteoarthritis and the need for surgical intervention later in life^16^. Moreover, research has indicated that early detection of an abnormal CAI through AP radiography can significantly improve patient outcomes by guiding timely and appropriate interventions, such as bracing or surgical correction^17^. Thus, the CAI in the AP view serves as a crucial parameter in the early diagnosis and management of AD, offering a predictive advantage over traditional bony indices alone.

This study aimed to evaluate the predictive importance of the CAI in diagnosing AD and to compare its accuracy with that of the traditional AI. By analyzing a cohort of patients with varying degrees of AD, this research sought to determine whether the CAI can serve as a reliable early marker for this condition and whether it can be integrated into routine clinical practice for the assessment of acetabular development.

## Methods

### Study design

In this study, 58 hips of 39 patients, 35 female and four male, who underwent surgery with intraoperative arthrography at the Dicle University Faculty of Medicine Orthopedics and Traumatology Clinic between 2011 and 2015 due to developmental dysplasia of the hip and had a follow-up period of at least 24 months were included in the study and examined retrospectively.

Patients with teratological hip dislocation, a follow-up period of less than 24 months and inadequate follow-up were not included in the study. Routine systemic examinations, blood group determination and blood counts were performed for all patients before surgery, and anesthesia opinions were obtained. Patients with pathological findings in their systemic examinations and blood values were taken into surgery after consultation with other relevant departments.

**Figure 1:**
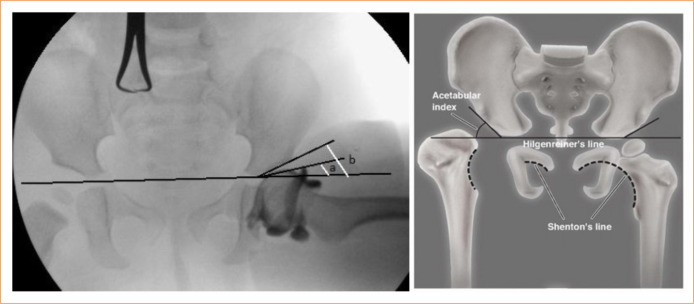
Radiologic view of the acetabular angle of the cartilage and schematic view of the acetabular index. **(a)** Cartilage acetabular angle. **(b)** Acetabular index.

Source: Elaborated by the authors.

Neutral pelvis AP radiographs were taken for preoperative radiological evaluation of the patients. Two of the patients had a history of using Pavlik bandage for different periods of time. All these patients underwent hip examinations before surgery. When measuring the bony acetabular angle, the lowest lateral point on the Y cartilage of the ilium was determined. Then, the most lateral point of the sclerotic part of the acetabulum was determined. The angle between the line connecting these two points and the line connecting both ilium points (Hilgenreiner) is the AI angle. The cartilage acetabular angle is the angle between the Hilgenreiner line drawn arthrographically after contrast material injection and the line drawn from the lowest lateral point on the Y cartilage of the ilium along the lower border of the cartilage acetabular roof (Fig. 1). In our study, patients whose cartilage acetabular angle was measured below 8º were determined as group 1, and those who were 8º and above were determined as group 2. In order to determine the importance of the CAI, both groups were compared in terms of bony acetabular development and secondary dysplasia development in patients with a follow-up period of at least 24 months.

No patient underwent skeletal or skin traction during the preoperative period. Among the 58 hips operated on for developmental hip dysplasia (DHD), 27 underwent medial limited open reduction, 15 underwent open reduction, one underwent adductor tenotomy and closed reduction, and 15 underwent only closed reduction.

The patients were evaluated for their radiological preoperative, early postoperative, and postoperative 24th month control bone AI angles, and intraoperative CAI angles after arthrography, and their secondary acetabular intervention requirements were assessed. Nineteen of the patients had bilateral DHD, 13 had left DHD, and seven had right DHD.

### Operation technique

The patients were operated on under general anesthesia and placed in the supine position, and the ipsilateral hip, pelvis and entire lower extremity were appropriately painted and prepared with batticon to allow free movement of the extremity during surgery. Arthrography was first performed on the patients. Median, subadductor access was preferred under scope. The needle was inserted just below the adductor longus, 2 cm distal to where it exited, and directed medially, targeting the contralateral sternoclavicular joint. One cc of physiological serum was injected to ensure that the joint was entered. After observing that the physiological serum returned under pressure, 1-2 cc of contrast agent was injected. The needle was removed after the contrast medium flowed freely around the femoral head. Radiographs were taken in each important position of the hip. It is important to determine the positions of maximum stability and instability^18^.

According to the arthrography results, open reduction, limited open reduction or adductor tenotomy were performed in patients in whom stable concentric reduction could not be achieved with closed methods. Afterwards, arthrography was performed again without opening the joint capsule. Intraoperatively, surgical options were decided during the operation according to whether concentric hip reduction could be achieved and age of the patients. Tönnis classification was used for arthrographic staging during surgery^19^. The goal of treatment was to obtain a Tönnis stage I hip:

- Stage I: The femoral head is completely seated and has come very close to the ischial part of the acetabulum;
- Stage II: The femoral head is under the acetabulum, but it is lateralized due to tension in the capsule, labrum and transverse acetabular ligament;
- Stage III: The femoral head is not under the labrum and is outside the acetabulum.

Tönnis stage I hips with a medial pooling of less than 2 mm were considered to have concentric reduction. The subcutaneous and skin were sutured with absorbable sutures, and a sterile dressing was applied. Afterwards, a pelvipedal cast was applied in the human position (hip in 90º of flexion and a maximum of 45–50º of abduction), and the operation was concluded.

The parents were taught postoperative patient care in a cast. None of the patients required intraoperative and postoperative blood transfusion.

### Postoperative care and follow-up

Until the first check-ups were made and the sutures were removed, the wound site was not opened, and no dressing was applied. After the patients’ pelvipedal casts were removed in the third postoperative month, an abduction device was applied continuously for three months and at night for three months.

The patients were called for monthly check-ups for the first three months postoperatively. During the periodic check-ups, the hip joint range of motion was examined in the clinical examination, and walking characteristics and pain were evaluated. Pelvic AP radiographs were taken to check the continuity of the Shenton line, the AI angle, the shape and change of the teardrop figure (Fig. 1).

### Radiological evaluation

In the radiological evaluation, the hips were evaluated in terms of AI angle change, secondary dysplasia and the need for secondary acetabular intervention. The AI angle was measured in the patients’ last follow-up radiographs, and the continuity of the Shenton-Menard line was checked (Fig. 1). The patients were followed up according to the parameters in the follow-up form, a copy of which is given below. In addition, postoperative complications such as re-dislocation, subluxation, infection, femur fracture, and avascular necrosis were checked.

The need for secondary acetabular intervention was determined with the secondary AD criteria. These criteria were:

- Fracture of the Shenton-Menard line;
- Acetabular index 32º or higher two years after reduction.

Mean, standard deviation, minimum and maximum values were given as descriptive statistics between the compared angular values (preoperative, intraoperative, and postoperative 24th month AI angles).

In this study, analyses were performed using unpaired t-test between dependent groups and non-parametric χ2 tests in the statistical evaluation of categorical variables. A 95% confidence interval was applied in all tests, and results were considered statistically significant for *p* < 0.05.

## Results

The youngest patient was 6 months old, the oldest was 30 months old, and the mean age was 14.33 months. The minimum follow-up period was 24 months, the maximum was 40 months, and the mean follow-up period was 28.23 months. The intraoperative CAI angles of the patients showed statistically significant changes according to the bone AI angles and the Shenton-Menard line fracture observed on the 24th month postoperative radiographs for secondary AD (*p* = 0.006, *p* = 0.033). Grade I avascular necrosis was seen in one of 39 hips. No additional complications such as re-dislocation, infection, or femur fracture were observed.

In this study, 16 of 58 hips (27.5%) required secondary acetabular intervention. When the relationship between preoperative AI and secondary AD was examined (Table 1), it was seen that one of 22 patients with a preoperative AI below 32º had secondary AD, and 16 of 36 patients with a preoperative AI of 32º and above had secondary AD, and the relationship between them was statistically significant (*p* = 0.001).

**Table 1:** Comparison of preoperative acetabular index and secondary acetabular dysplasia.

		Available	Absent	Total
Preoperative acetabular index	32º <	1	21	22
	32º ≥	16	20	36
Total		17	41	58
*p* = 0.001				

Source: Elaborated by the authors.

When the intraoperative CAI angles of the patients were evaluated according to the bone AI angles (Table 2) observed on the postoperative 24th month radiographs for secondary AD, it was observed that two out of 25 patients in group 1 had postoperative 24th month bone AI angles of 32º and above, and 14 out of 33 patients in group 2 had postoperative 24th month bone AI angles of 32º and above, and there was a statistically significant relationship between them (*p* = 0.006).

**Table 2:** Intraoperative and postoperative 24th month acetabular index comparison.

	32º <	32º ≥	Total
Group 1	23	2	25
Group 2	19	14	33
Total	42	16	58
*p* = 0.006			

Source: Elaborated by the authors.

When the intraoperative CAI of the patients was evaluated according to the fracture in the Shenton-Menard line for secondary AD (Table 3), one out of 25 patients in group 1 had a fracture in the Shenton-Menard line, while nine out of 33 patients in group 2 had a fracture in the Shenton-Menard line, and there was a statistically significant relationship between them (*p* = 0.033).

**Table 3:** Intraoperative comparison of cartilage acetabular index and Shenton-Menard line.

	Unbroken bone	Bone fracture	Total
Group 1	24	1	25
Group 2	24	9	33
Total	48	10	58
*p = 0.033*			

Source: Elaborated by the authors.

After three months of postoperative plaster application, all patients used abduction devices regularly. Although the mean follow-up period was insufficient to determine the rate of avascular necrosis, grade I avascular necrosis was seen in one of 39 hips. None of the patients had re-dislocation, subluxation, neurovascular damage, or infection. No intraoperative or postoperative blood transfusion was required.

## Case presentations

**Figure 2:**
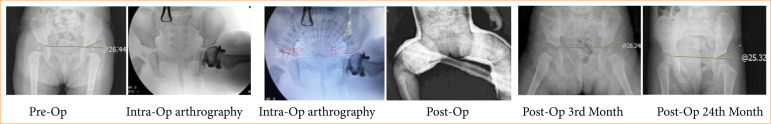
Sixteen months old, female, left developmental hip dysplasia, preoperative acetabular index: 26º, cartilage acetabular index: 5º, no dysplasia developed.

Source: Elaborated by the authors.

**Figure 3:**
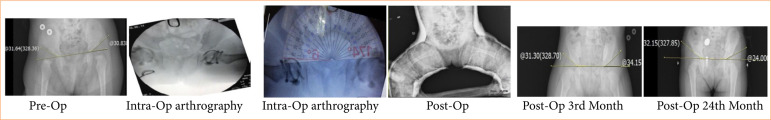
Ten months old, female, bilateral developmental hip dysplasia, right preoperative acetabular index: 31º, left preoperative acetabular index: 30º, cartilage acetabular index right: 17º, left: 6º, dysplasia developed on the right side, no dysplasia developed on the left.

Source: Elaborated by the authors.

**Figure 4:**
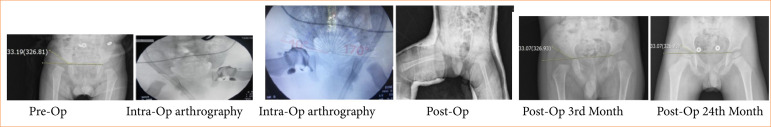
Fourteen months, male, right developmental hip dysplasia, preoperative acetabular index: 33º, cartilage acetabular index: 10º, dysplasia developed.

Source: Elaborated by the authors.

Three cases showing the pre-operative, intra-operative and post-operative processes of our study are presented in full detail (Figs. 2, 3 and 4).

## Discussion

The CAI has emerged as a significant advancement in the assessment of AD, offering a nuanced approach that complements traditional radiographic measures. Historically, the diagnosis and management of AD have relied heavily on bony indices such as the AI and the LCEA. While these measures have been instrumental in identifying dysplasia, they primarily assess the osseous components of the acetabulum, often overlooking crucial cartilaginous factors.

Recent advancements in imaging technology, particularly MRI, have facilitated the evaluation of CAI, which incorporates both the cartilaginous and bony aspects of the acetabulum. This comprehensive approach has the potential to enhance diagnostic accuracy and predictive capabilities in orthopedic surgery. The CAI’s ability to detect subtle cartilaginous abnormalities before they manifest as significant bony changes is particularly valuable in early diagnosis and treatment planning. Moreover, the integration of CAI into clinical practice has implications for surgical strategies, allowing for more precise planning and potentially improving long-term patient outcomes.

As the field of orthopedic surgery continues to evolve, understanding the predictive importance of CAI in the context of AD becomes increasingly critical. This discussion explored the role of CAI in preoperative assessment, its impact on surgical decision-making, and its prognostic value in predicting long-term outcomes, highlighting its advantages over traditional diagnostic methods and addressing current challenges and future directions in its application.

The predictive value of CAI in detecting AD is increasingly recognized in recent research. Unlike traditional indices such as the AI and LCEA, which primarily assess bony structures, CAI evaluates the cartilaginous component of the acetabulum^20^. This distinction is particularly important in pediatric populations and early stages of acetabular development, in which cartilage plays a critical role. CAI provides a more sensitive measure of dysplastic changes by capturing deviations in the cartilaginous acetabular rim that may not be evident through traditional radiographic assessments^5^.

Studies have shown that incorporating CAI into the diagnostic process enhances the early detection of dysplasia, allowing for timely intervention and potentially reducing the progression to more severe forms of the condition^21^. In our study, a significant relationship was found between the criteria for secondary AD in 14 of 33 patients with a cartilage acetabular angle of 8º and above, and secondary AD developed in only two of 25 patients with an angle below 8º.

The integration of CAI into preoperative planning has demonstrated considerable benefits. MRI is the primary imaging modality used for CAI measurement, offering high-resolution images that detail both cartilaginous and bony structures of the acetabulum^22^. This detailed visualization is crucial for surgical planning, particularly in complex procedures such as periacetabular osteotomy and total hip arthroplasty. Accurate assessment of CAI allows surgeons to better understand the degree of dysplasia and the specific cartilaginous abnormalities that need to be addressed. This, in turn, enables more precise surgical interventions, such as the optimal positioning and alignment of the acetabulum, which is essential for achieving favorable outcomes and reducing the risk of postoperative complications^23^.

In our study, arthrography was performed again without opening the joint capsule. Tönnis classification was used for arthrographic staging during surgery^19^. The goal of treatment is to obtain a Tönnis stage I hip. The decision to achieve concentric reduction and a stable hip should be made with arthrography. The quality of the reduction cannot be assessed without arthrography, and, during arthrography, the hip should not be forced for reduction, and reduction should be achieved only with hip flexion and abduction.

The long-term implications of CAI measurements are profound. Research has established a strong correlation between abnormal CAI values and the risk of developing osteoarthritis, highlighting CAI’s role in predicting long-term joint health^24^. Patients with elevated CAI values are at a higher risk for progressive joint degeneration and may require earlier and more aggressive interventions to prevent the development of severe osteoarthritis. This predictive capability underscores the importance of incorporating CAI into routine assessments to guide treatment decisions and optimize patient management strategies^8^.

CAI also holds promise for postoperative monitoring. The ability to track changes in the cartilaginous acetabulum over time allows for continuous evaluation of surgical outcomes and the detection of any recurring dysplastic changes^25^. This ongoing assessment is crucial for adjusting treatment plans and ensuring that any adverse developments are addressed promptly. For instance, if postoperative CAI measurements indicate persistent or worsening dysplasia, additional interventions may be warranted to maintain joint function and prevent further deterioration^26^. In this study, patients were followed up monthly for the first three months. Hip joint range of motion, mobility, and pain were evaluated in periodic check-ups. Also, the monitored parameters were evaluated with pelvic AP radiographs.

Despite its advantages, CAI has certain limitations that need to be addressed. MRI, while highly effective, is not always accessible or cost-effective for all patients, particularly in resource-limited settings^27^. The variability in measurement techniques and interpretation can also impact the reliability of CAI values. To enhance the utility of CAI in clinical practice, there is a need for standardized measurement protocols and further research into alternative imaging techniques that can provide similar levels of accuracy at a lower cost^28^.

Future research should focus on validating CAI’s effectiveness across diverse patient populations and different clinical scenarios. Additionally, exploring the integration of CAI with other diagnostic and predictive tools could further enhance its role in the comprehensive management of AD^29^. Investigations into the long-term outcomes associated with CAI-guided interventions will be also crucial in establishing its definitive role in improving patient prognosis and optimizing surgical results. In our opinion, the shortcomings of our study are the difference in age distribution, the lack of homogeneity in the variety of treatment methods, and the short follow-up period. Studies with homogeneous treatment methods and longer follow-up periods are needed to investigate the importance of the cartilage acetabular angle.

## Conclusion

The CAI represents a significant advancement in the assessment of AD. Its ability to provide a detailed evaluation of the cartilaginous acetabulum, combined with its predictive value for long-term outcomes, makes it a valuable tool in orthopedic surgery. By integrating CAI into clinical practice, surgeons can enhance their diagnostic accuracy, improve surgical planning, and achieve better long-term results for patients with AD. Continued research and refinement of CAI measurement techniques will further solidify its role in optimizing patient care and outcomes.

## Data Availability

All data sets were generated or analyzed in the current study.
